# Influence of the Binder Jetting Process Parameters and Binder Liquid Composition on the Relevant Attributes of 3D-Printed Tablets

**DOI:** 10.3390/pharmaceutics14081568

**Published:** 2022-07-28

**Authors:** Klemen Kreft, Zoran Lavrič, Tijana Stanić, Petra Perhavec, Rok Dreu

**Affiliations:** 1 Faculty of Pharmacy, University of Ljubljana, Aškerčeva Cesta 7, 1000 Ljubljana, Slovenia; klemen.kreft@ffa.uni-lj.si (K.K.); zoran.lavric@ffa.uni-lj.si (Z.L.); 2 Lek Pharmaceuticals d.d., a Sandoz Company, Verovškova Ulica 57, 1000 Ljubljana, Slovenia; tijana.stanic@sandoz.com (T.S.); petra.perhavec@sandoz.com (P.P.)

**Keywords:** binder jetting, design of experiments, 3D-printed tablets, mechanical properties, printer development, ketoprofen

## Abstract

Binder jetting has the potential to revolutionize the way we produce medicine. However, tablets produced by binder jetting technology can be quite fragile and hard to handle. In this study, the printing process and ink composition were examined to optimize the mechanical properties of tablets. A model formulation containing the ketoprofen drug was developed and used as a base for optimization. Firstly, important printing parameters were identified with a fractional factorial design. Saturation and layer height critically influenced selected tablet properties. Relevant process parameters were optimized for tablet mechanical strength by using the D-optimization DoE approach. The best mechanical properties were achieved when saturation was set to 1 and layer height to 150 µm. On the other hand, binder ink composition did not appear to impact tablet mechanical strength as much as process parameters did. Three ethanol-water mixtures were tested at three tablet strength levels and no definitive conclusions could be drawn. The binder jetting process can be wasteful, especially if the unbound powder cannot be reused. To determine the suitability of powder blend recycling, the ketoprofen content was measured for 27 subsequent batches of tablets. While the trendline did indicate a slight reduction in ketoprofen content, the powder blend reuse can nevertheless be employed.

## 1. Introduction

Since patient-oriented care was proposed, the health care industry has been trying to develop new technologies to tailor medication to the individual patient. Substantial research efforts are being made in an attempt to solve the puzzle of manufacturing, testing, and delivering tailored medications. Particularly with solid dosage forms, custom manufacturing can be challenging because dosage forms are uniform and generally not designed to be divided for the purpose of personalized dosing. Conventional manufacturing technologies may be less suitable for the production of individualized medicines. Nevertheless, growing demand for tailored therapy is forcing researchers to explore new avenues. The use of additive manufacturing or 3D printing of dosage forms has been proposed for the development of pharmaceutical products. The first 3D printing technology dates back to 1986 [1]. However, it has only recently gained enough traction due to technological advances.

The 3D printing of tablets has several advantages over conventional manufacturing, such as the possibility of individualized dosing for each patient through on-demand manufacturing by specialized contract manufacturers, customized shape for easier swallowing, the inclusion of more active ingredients in a single polypill, precise dosing of potent active ingredients, etc. [2,3]. All of these can lead to higher adherence, fewer side effects, and greater efficacy of dosage forms. For pediatric patients, rare diseases, or geriatric patients, accurate personalized dosing can be assured, increasing the appropriateness of each treatment [4]. There are different technological branches within the 3D printing technology: fused deposition modelling (FDM), selective layer sintering (SLS), stereolithography (SLA), semi-solid extrusion (SSE), and binder jet 3D printing, to name a few. The latter technology was used by Aprecia Pharmaceuticals to develop the first 3D-printed drug Spritam (levetiracetam), which was approved by the FDA in August 2015. The production of Spritam demonstrates that binder jetting can be used on a large scale [3].

Binder jetting is a layer-by-layer manufacturing process in which powder is first distributed in a fine layer. The particles are then bound together by a binding fluid applied via an inkjet printhead. This process continues over many layers until the final dosage form is produced. A final curing step is required to remove the solvent and fully solidify the layers, usually with convection ovens or IR light. Binder jetting has been used to produce tablets with immediate release [5,6] or more complex kinetics such as pulsatile [7], as well as delayed [8] and zero-order release profiles [9]. Different binding fluids can be applied to each part of the tablet, resulting in complex core-shell structures with different properties [6,10].

One of the main characteristics of such tablets is their high porosity. Most reported porosity values range between 50 and 60% [11,12,13,14], in certain cases even 80% [5,15]. In binder jetting, the particles are bound and held in place only by the binding liquid. This contrasts with conventional tableting, where high compressive forces are involved in order to form molecular interactions and solid bridges. Consequently, 3D-printed tablets produced by binder jetting can have a disintegration time of a few seconds. This feature is particularly desirable for adherence-related high-risk patient groups or patients with swallowing difficulties. On the other hand, high porosity and larger spacings between particles lead to high fragility and low tablet hardness. These properties are not desirable because they make the tablets difficult to package, transport, and handle. This can be avoided by implementing formulation approaches, which have been extensively studied. To achieve good tablet strength, the selection of appropriate excipients is crucial [11].

When evaluating the processability of powder mixtures in terms of binder jetting technology, flowability, wettability, and consolidation of particles seem to be most important [15,16]. These can be easily measured with established pharmaceutical methodology. From a material point of view, the binding liquid alone seems to bind the particles together rather poorly. It is advantageous to add a soluble solid binder to the powder blend to improve particle binding, increase tablet hardness, and augment liquid uptake. A soluble solid binder is activated in situ in the presence of liquid and binds neighboring particles into a strong compact [15]. The powder mixture should also allow for rapid liquid absorption, ideally within 2 s. This is determined by high wettability [17]. To ensure good particle linkage, the liquid should penetrate into the layer and activate the solid binder. Vertical liquid penetration is critical [16]. Droplets should not remain on the surface, as this can lead to unnecessary surface defects, bad layer interlinkage, and poor spreadability of the powder in the next layer. To better understand vertical penetration, a dimensionless number, the degree of overlap, was defined as a ratio of penetration depth of the liquid binder to layer thickness. It affects the mechanical properties of tablets [14].

Binding liquid processability and deposition repeatability are also critical. Ink properties describing processibility are viscosity, surface tension, and density. These properties are represented by the Ohnesorge number, a dimensionless number [18]. Each printhead is, of course, optimized for different ink properties. However, there is a general consensus that an Ohnesorge number between 0.1 and 1 allows for at least some degree of binding fluid processability by printing nozzles [18]. Lower values could result in unnecessary satellite droplets, while higher values could hinder droplet deposition from the nozzle due to high viscous forces [19]. The binding liquid can also include drug particles as a solution or suspension. However, care should be taken to prevent nozzle clogging, since common reported nozzle diameters of printheads are usually below 50 µm [5,8,10,11,12]. In addition, pigments can be added effortlessly to color-specific parts of the tablet or to the entire tablet [13].

The process of binder jetting has been simulated on a smaller scale for formulation purposes. Sen et al. have developed a representative drop test to test various powder blends for their suitability for binder jetting. The drop test resembles the printing process on a small scale and involves a single drop of binding fluid onto a powder bed in a Petri dish. Properties of resulting compacts can be evaluated with a texture analyzer and imaging techniques [20].

There is still a scarce number of publications dealing with the binder jetting printing process and the influence of the process parameters on the properties of the produced tablets. Apart from the binder saturation level, layer thickness, and powder spreading rate, the process parameters for pharmaceutical binder jetting have been largely neglected. Lower layer heights and a higher degree of binder saturation did result in firmer tablets that are easier to handle [14,21]. Nevertheless, there are other possible crucial process parameters worth investigating. In this study, we analyzed the printing process using an in-house developed Picojet pharmaceutical 3D printer and looked for relationships between different printing parameters and properties of finished tablets.

In addition, we investigated the influence of the composition of the binding liquid on the mechanical properties of the tablets. Solvent selection could be important from the formulation standpoint to optimize the mechanical properties of 3D-printed tablets for easier handling. Once the binding liquid is applied, the solid binder becomes dissolved in situ and activates. Before the binder dries, insoluble particles are primarily held together by the maximum capillary pressure, which is proportional to γL × cosΘ, where γL is the surface tension of the binding liquid and Θ is the contact angle [22]. In theory, an increase in the product would stem from higher capillary pressure, resulting in stronger connections between particles. Since both parameters can be easily measured, this method could potentially serve as a good screening tool to optimize the ink composition for each powder blend. Changing ink’s composition should influence its ability to bind particles together via liquid bridges due to changed wettability of solid particles and changed surface tension.

## 2. Materials and Methods

### 2.1. Powder Bed Materials and Blending

The active pharmaceutical ingredient ketoprofen and all other excipients were assured from Lek d.d. stock. A number of pharmaceutical grade fillers were first screened for processability determination. Pharmatose^®^ 200M (lactose monohydrate), Pharmatose^®^ 125M (lactose monohydrate), and Supertab^®^ 14 SD (spray-dried lactose monohydrate) were sourced from DFE Pharma (Goch, Germany), Avicel PH-101^®^ (microcrystalline cellulose) from Dupont (Wilmington, DE, USA), Tabletosse^®^ 70 (lactose monohydrate) from Meggle GmbH & Co. KG (Wasserburg am Inn, Germany), and Pearlitol^®^ 100 SD (mannitol) from Roquette Frères (Lestrem, France). A solid binder was added to the powder blend, as this option allows for more robust dosing of pure liquid solvent via the printhead instead of introducing a dissolved binder via the binding liquid. The binder Plasdone^®^ K-25 (polyvinyl pyrrolidone grade K 25) was sourced from Ashland (Wilmington, DE, USA). To improve flowability of the powder blend and facilitate powder deposition in layer form, a silica-based glidant was added to the powder mixture. The glidant Syloid^®^ 244 FP (silica) was sourced from Grace GmbH (Worms, Germany). Final powder blends were composed of 20.0% ketoprofen, 69.5% filler, 10% Plasdone^®^ K-25, and 0.5% of Syloid^®^ 244 FP.

Ketoprofen was sieved through a 200 µm mesh sieve to remove large particles and equate particle size with other excipients. In this way, potential particle segregation was prevented. All other excipients were sieved through a 315 µm mesh sieve to remove larger lumps and agglomerates. Powder blends were mixed in a 2 L tumbler mixer (Inversina, BioEngineering, Walt, Switzerland) for 5 min at a mixing intensity of 3.5.

### 2.2. Binding Liquid Preparation

Since water has high surface tension, it can be difficult to atomize during the binder jetting process [17]. This was also confirmed in our preliminary studies; the printhead could not effortlessly apply water. Therefore, ethanol-water mixtures with lower surface tension were explored for process suitability. All ethanol-water mixtures possess viscosity below 3 mPas [23]. To improve processability, a low molecular weight thickener was added to increase the viscosity. The thickener Polyglykol 1500 S (polyethylene glycol 1500) (PEG) was donated by Clariant AG (Muttenz, Switzerland). For screening purposes, 10, 20, 30, and 40 m/m % ethanol-water mixtures without PEG were prepared. Once mixture properties were determined and initial printing processibility assessed, 10% of PEG was added to the processable solutions. Inks were mixed with a magnetic stirrer until PEG completely dissolved.

### 2.3. Particle Size and Flowability of Powder Blends

The flowability of powder blends was assessed through bulk and tapped densities, as described in the European Pharmacopoeia, 10th edition, 2020; 2.9.34. Bulk density and tapped density of powders. Each powder blend was gently introduced into a 100 mL measuring cylinder. Samples were tapped 1250 times (VanKel Tap Density Tester; Varian Inc., Cary, NC, USA) in triplicates. Subsequently, the Carr index (CI = 100((ρ_t_ − ρ_b_)/ρ_t_) and Hausner ratio (HR = ρ_t_/ρ_b_) were calculated, where ρ_t_ and ρ_b_ are tapped and bulk density, respectively.

Powder mixture particle size distribution may limit the selection of certain printing parameters, such as the thickness of each printed layer. The D90 should be lower than the layer thickness, so that the particles can be evenly spread into the proper thickness [15]. The laser diffraction technique (Mastersizer S; Malvern Panalytical Ltd., Malvern, UK) was used to assess the particle size distribution of all solid raw materials. D10, D50, and D90 denote 10, 50, and 90% of the cumulative undersize particle size distribution. Measurements were carried out in a dry cell with a dry dispersion unit operating at 0.3 bar. Each sample was measured four times and average values of percentiles were calculated.

### 2.4. Properties of Binding Fluids

To determine printing suitability of various ethanol-water mixtures, viscosity, surface tension and density of liquids were measured beforehand. Viscosity was evaluated by using a Physica MCR 301 rheometer (Anton Paar, Graz, Austria) with a CC27 bob and cup system temperated at 25.0 °C. Shear rate gradually changed from 1 s^−1^ to 100 s^−1^ during the rotational tests for a total duration of 200 s. Surface tension measurements were assessed by the Wilhelmy plate method using a standard platinum plate (Tensiometer K12, Kruss GmbH, Hamburg, Germany). All measurements were carried out at 25.0 °C. Binder liquid density was determined with a Guy Lussac pycnometer relatively to water. The temperature of all samples was maintained at 25.0 °C with a water bath and was checked with an external thermometer before measuring.

### 2.5. Contact Angle

The sessile drop method was used to measure the contact angle between powder blends and various binder liquids. The powder blend was first compressed into compacts (approximately 200 mg) by a circular steel punch and die assembly (d = 13 mm) and a Specac hydraulic press (Specac Ltd., Kent, UK) with a 10 s dwell time at a force of approximately 49 kN. No surface defects of compacts were observed. The contact angle was measured with a drop shape analyzer Krüss DSA100 (Krüss GmbH, Hamburg, Germany). Each binder liquid was added to the sample surface in the form of 5 µL drop and the contact angle was measured with a circle-fitting method in 12 replicates, and average value was reported.

### 2.6. The Picojet D220 3D Printer

**Figure 1:**
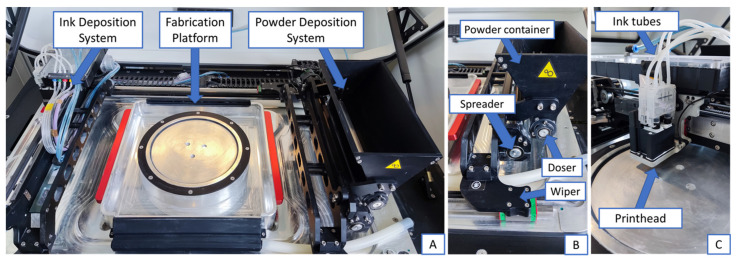
Key components of the Picojet D220 3D printer. The main parts are displayed in (**A**), the powder deposition system in (**B**) and the ink deposition system in (**C**).

The Picojet D220 (Spectra Laboratories, Ljubljana, Slovenia) is a custom-developed 3D printer for binder jetting of pharmaceutical products. It is composed of three main parts: the ink deposition system, the powder deposition system, and the fabrication platform (Figure 1A). The general binder jetting principles apply. The powder deposition system spreads the unbound powder into a thin layer. Then, the printhead activates and applies the binding liquid onto the powder bed to connect the particles together and form the first layer of the tablet. Before the second layer is prepared, the fabrication platform descends to create room for the new layer. This descending move determines the thickness of each layer, defined as layer height. The process continues in several layers until the tablet volume is completed. Once printing is finished, a drying step is necessary to remove excessive solvents and solidify the in situ dissolved solid binder.

The powder deposition system includes three elements: the doser, the spreader, and the wiper (Figure 1B). Between 500 and 2000 g of powder can be manually added to the powder container. Dosing is executed by the doser, a dosing cylinder at the bottom of the powder container where particles can exit through a 2 mm gap. The doser spins in a counterclockwise direction with the maximum circumferential speed of 4 mm/s. Once the powder is applied to the fabrication platform, the spreader (a spreading cylinder) activates to spread the powder into a fine layer. The spreader also removes any excess powder from the fabrication platform with the maximum circumferential speed of 40 mm/s. Excess powder is collected around the fabrication platform and can be reused in subsequent batches. The powder deposition system is mounted to the so-called wiper, a motorized arm passing the fabrication platform. The wiper regulates the time available for the doser and spreader to create a uniform layer. Maximum wiper speed is set to 120 mm/s.

The primary component of the ink deposition system is the printhead (Figure 1C). The piezoelectric printhead is comprised of 1280 nozzles with approximately 20 µm inner nozzle diameter size. It can process 4 different inks simultaneously, since 320 nozzles are dedicated to each ink. Printhead resolution is 300 × 1200 DPI per selected binding fluid. Printhead lateral speed can be accurately controlled up to a maximum of 150 mm/s. Each nozzle can be selectively activated or deactivated to set the amount of jetted ink in each pass, defined as nozzle saturation level parameter or simply saturation. It can be varied between 0 and 100%. If saturation is set to 100%, ink is ejected twice from each nozzle and at 50%, ink is applied in two subsequent droplets from half of the nozzles uniformly distributed on the pixel level.

### 2.7. 3D Printing Experiments

For this series of experiments, 2000 g of the powder blend was manually loaded into the powder container. Any excess powder removed by the spreader was reused for the next tablet batches. The binding fluid was filled into a 1 L reagent bottle which is connected to the printhead via four silicone tubes. Round tablets were designed in the SpectreSlicer software (Spectre Laboratories, Ljubljana, Slovenia) with a 15 mm diameter and 5 mm height. A series of 40 tablets were printed per each batch with 5 mm spacing between tablets for easier manual removal from the fabrication platform. Printing parameters such as printhead speed, layer height, doser speed, saturation level, and spreader speed were set to specific values according to experimental design, described in Section 2.8. Once the tablet design and printing parameters were set, information was transferred to the 3D printer via a USB stick and 3D printing started. Before the binding liquid was jetted, the powder deposition system applied several layers of powder of around 1 cm thickness to prevent the adhesion of the first layer to the fabrication platform. Once the printing process finished, samples were manually removed from the fabrication platform and put into a convective drying chamber (Kambič, Semič, Slovenia) for 150 min at 60 °C to dry to loss on drying (80 °C, 30 min) values below 2%.

### 2.8. Design of Experiments

The design of experiments (DoE) was created in software Modde 12.01 (Sartorius, Göttingen, Germany). An optimal number of experiments was proposed to study the influence of 5 process parameters on selected tablet responses. Since binder jetted tablets can experience poor mechanical properties, hardness, friability, and disintegration time were identified as suitable responses. In addition, mass, mass uniformity, and tablet dimensions were examined since they represent important tablet properties. We were investigating 5 process parameters: spreader speed, doser speed, layer height, printhead speed, and saturation level.

The DoE was planned in two stages. First, a screening phase was prepared to single out impactful process parameters. Two-level fractional factorial design was executed with three additional central points to evaluate the reproducibility of results. A total of 11 experiments were carried out (N1–N11). Subsequently, an optimization phase with a D-optimized design approach was initiated to investigate interactions between impactful parameters and create a model with higher prediction ability. Seven additional experiments were performed (C12–C18). The process parameter settings of the screening and optimization phase experiments are described in Table 1. Appropriate levels of processing parameters were determined based on preliminary studies. Layer height could not be increased above 250 μm, as otherwise layers were poorly interlinked, and tablets were not strong enough to be handled. Doser speed could not be reduced below 3 mm/s since powder deposition was insufficient, and powder layer was not uniformly formed. Wiper speed was set to 60 mm/s and was kept constant. All DoE experiments were performed with a 20% ethanol-water mixture as the binder liquid.

### 2.9. Binding Fluid and Tablet Mechanical Properties

To assess the influence of ink composition on the mechanical properties of tablets, additional experiments were carried out with 10, 20, and 30% ethanol-water mixtures. This could only be accurately assessed if the applied volume was constant for all inks. However, the printhead forms droplets of different volumes for inks with different composition. Therefore, the amount of applied liquid and the volume of each droplet for every ink was evaluated. Jetted droplets were collected on a piece of plexiglass, placed below the printhead, and weighed after 1, 2, 5, 10, 20, 30, 40, and 50 jetting cycles. The volume of each droplet was calculated from Equation (1):

(1) $$V=\frac{m}{\rho \;\times \;n\;\times \;a\;\times \;p\;\times \;r}$$

where V is the volume of ink’s droplet, m is the mass of applied ink after jetting cycles, ρ is ink density, n is number of jetting cycles, a is number of active nozzles (a = 320), p is number of printhead passes (p = 2), and r is the number of concurrent replicates (r = 3).

The saturation of the printhead was then adjusted for each fluid so that the print-head applied the same volume for all three inks via a different number of active nozzles. In total, nine tablet printing experiments using three different binder liquids were carried out. The influence of ethanol content in ink was tested at anticipated three different tablet mechanical strength levels. These were defined by the process parameters settings (Table 2). Doser speed was set to 3.5 mm/s, spreader speed to 30 mm/s, printhead speed to 90 mm/s, and wiper speed to 60 mm/s.

### 2.10. Analysis of Tablets

**Figure A1:**
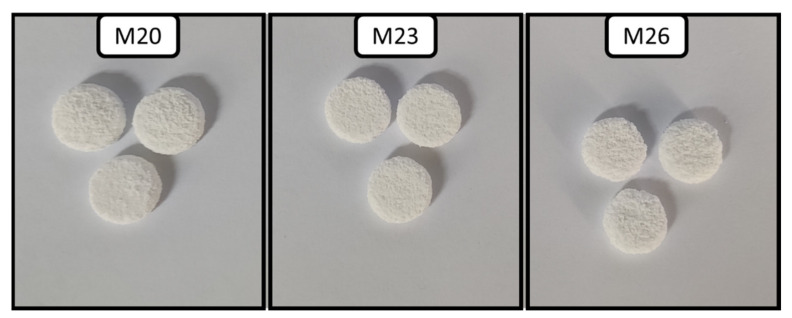
Visual representation of 3D-printed tablets from experiments M20 (high strength), M23 (medium strength), and M26 (low strength) with the 20% ethanol-water mixture. As can be discerned, an excess of material at tablet edges is observed for experiment M20, while experiment M26 yielded poorly bonded tablets due to high layer height and low saturation.

Prepared tablets (examples depicted in Figure A1) were analyzed for dimensions, mass, mass uniformity, hardness, friability, disintegration, and ketoprofen content. Tablet diameter and height were measured with a digital beak gauge (Unior d.d., Zrece, Slovenia) in 10 replicates and an average was calculated. Tablet mass was determined on an analytical scale (Sartorius AX224, Sartorius AG, Göttingen, Germany) in 10 replicates and an average mass and relative standard deviation were calculated. Hardness was evaluated on a Kraemer HC 97 tablet hardness tester (Kraemer Elektronik, Darmstadt, Germany) in 5 replicates, as described in the European Pharmacopoeia, 10th edition, 2020; 2.9.8 Resistance to crushing of tablets. Friability was measured on a TAR friability tester (Erweka GmbH, Langen, Germany) as described in the European Pharmacopoeia, 10th edition, 2020; 2.9.7. Friability of uncoated tablets. Drum rotational speed was set to 25 rpm for 2 min. Tablet disintegration times were evaluated by performing a disintegration test carried out in a DISI-2M disintegration tester (Charles Ischi AG, Zuchwil, Switzerland) according to the European Pharmacopoeia, 10th edition, 2020; 2.9.1. Disintegration of tablets and capsules. Ketoprofen assay in tablets was assessed with an Agilent 8453 UV/VIS spectrophotometer (Agilent Technologies, Santa Clara, CA, USA). Five tablets per sample were crushed with a mortar and pestle. Approximately 1000 mg of powder (around 200 mg of ketoprofen) was transferred into a 200 mL flask and dissolved in 80 *v/v* % acetonitrile (ACN) solution (c_keto_ ≈ 1000 mg/L). Samples were put in an ultrasonic bath for 30 min before further dilution (c_keto_ ≈ 10 mg/L) and filtration (0.45 μm RC 25 filter). Ketoprofen assay was measured at 254 nm wavelength and a calibration curve was prepared with R^2^ = 0.99966.

## 3. Results

### 3.1. Powder Formulation Studies

Several fillers were screened to determine the processability of final powder blends from the flowability and particle size point of view (Table 3). Seven powder mixtures (Z1–Z7) were prepared and powder deposition was tested on the Picojet platform. The Z1 and Z2 did not exit the powder deposition system due to poor flow properties despite varying doser and wiper speed. The Hausner ratio was above 1.46 for both mixtures, indicating very poor flow character according to the Scale of Flowability from European Pharmacopoeia, 10th edition, 2020; 2.9.36. Powder Flow. On the other hand, Z4, Z5, and Z6 were freely oozing out of the powder deposition system, even when deposition was not necessary. This could create unnecessary material depositions on the powder layer and around the fabrication platform, which is not desirable. Powder blends Z4–Z6 were too flowable and might affect the quality of the final tablets. The gap between the doser and the powder container is too large for these mixtures with Hausner ratios below 1.26. Only Z3 and Z7 were correctly deposited from the powder deposition system. It appears that the most optimal flow character based on the Scale of Flowability is Passable and Poor, with a Hausner ratio at least between 1.26 and 1.37.

The thickness of each deposited powder layer is limited by the particle size distribution of the powder blend. Therefore, particle size distributions of raw materials were also measured. To accommodate the lowest planned layer height of 150 μm, the D90 of every component should not deviate too much from it. The D90 of fillers Tabletosse^®^ 70 and Supertab^®^ 14 SD was measured at 327 and 228 µm respectively, which would prevent the formation of a 150 µm layer. However, the D90 of Pearlitol^®^ 100 SD and Pharmatose^®^ 125 M were more suitable at 152 and 165 µm respectively. Due to pleasant mouthfeel of Pearlitol^®^ 100 SD and good powder deposition of Pharmatose^®^ 125 M, the final composition Z7 was selected for further printing experiments. In addition, the D50 of all major components in the Z7 mixture were similar. This indicates a uniform particle size distribution of the Z7 mixture, which could prevent ketoprofen segregation during and between printing runs. Throughout the trials, the same powder blend was reused several times. The absence of powder mixture segregation was crucial to maintaining the homogeneity of the powder blend.

### 3.2. Binding Fluid Formulation Studies

Repeatable and adequate binding fluid application is crucial for the printing process to succeed. Viscosity, density, and surface tension are the ink’s primary features evaluated in the formulation studies, while ink processability is depicted by the Ohnesorge number (Equation (2)):

(2) $$\mathrm{Oh}=\frac{\eta }{\sqrt{\gamma \;\times \;\rho \;\times \;a}}$$

where Oh is the Ohnesorge number, η, ρ, and γ are the dynamic viscosity, density, and surface tension of the binding fluid, and a is the diameter of the nozzle [18]. Ohnesorge numbers between 0.1 and 1 correspond to binder liquids processable by piezoelectric nozzles within the printhead [18]. This range of Oh values was the goal during our formulation development studies.

Based on the manufacturer’s guidelines, binding fluids with the following properties can be repeatably applied on the Picojet: density between 0.85 and 1.3 g/mL, surface tension between 20 and 40 mN/m, and viscosity between 2 and 14 mPas. 10, 20, 30, and 40% ethanol-water mixtures were prepared after initial screening and processability was qualitatively confirmed on the Picojet system. Solutions with higher or lower ethanol content could not be applied by the printhead as was determined in preliminary studies. While the density and surface tension were in line with the manufacturer’s guidelines, the viscosities of the initial solutions were below or close to the lower viscosity threshold for processability (Table 4). The Ohnesorge number was also calculated to be between 0.046 and 0.102, which could have led to satellite drop formation [18]. Therefore, 10% of PEG 1500 was added to the initial solutions to increase viscosity for around 2 mPas, and consequently to increase the Ohnesorge number. The addition of PEG did not significantly influence the density, surface tension, and contact angle of the binder liquids. However, these solutions were not processable due to the quick nozzle clogging phenomenon. When droplets were formed, PEG solidified, clogged the nozzles, and prevented droplet ejection. With increasing complexity of the ink’s composition, the possibility for nozzle clogging becomes substantial [12]. For this reason, PEG was omitted from the formulation and 10, 20, 30, and 40% ethanol-water mixtures were used in the continuation of the study. Despite the low Ohnesorge number, droplet ejection occurred as intended and no obvious satellite droplets or random ink oozing were observed.

Maximum capillary pressure is a measure of the liquid bridge strength between particles. It correlates with the product of the surface tension of the binding fluid and the cosine function of the contact angle between the binding fluid and the blend particle. The highest product of the γL × cosΘ could result in the strongest bonds between particles after drying, and the best mechanical properties of tablets [24]. The contact angle was therefore measured for all binding fluids with the Z7 powder blend. The best wettability was achieved between the 40% ethanol-water mixture and Z7, with the contact angle of 16.0° (Table 4). This means the 40% ethanol-water mixture spreads over and maintains the most contact surface with the particles of the Z7 powder blend. Wettability decreased proportionally with decreasing ethanol content. The contact angle for the 10% ethanol-water mixture was as high as 31.7°. Nevertheless, the product of γL × cosΘ was the highest for the 10% ethanol-water mixture due to higher surface tension. We therefore expected that the strongest bonds between particles will be formed after drying with the use of the 10% ethanol-water mixture. Proportionally increasing ethanol content decreased the product of γL × cosΘ, which might, at least in theory, lead to tablets with slightly poorer mechanical properties. This hypothesis was tested and the results are outlined in Section 3.4.

### 3.3. Influence of Process Parameters on Tablet Properties

**Figure 2:**
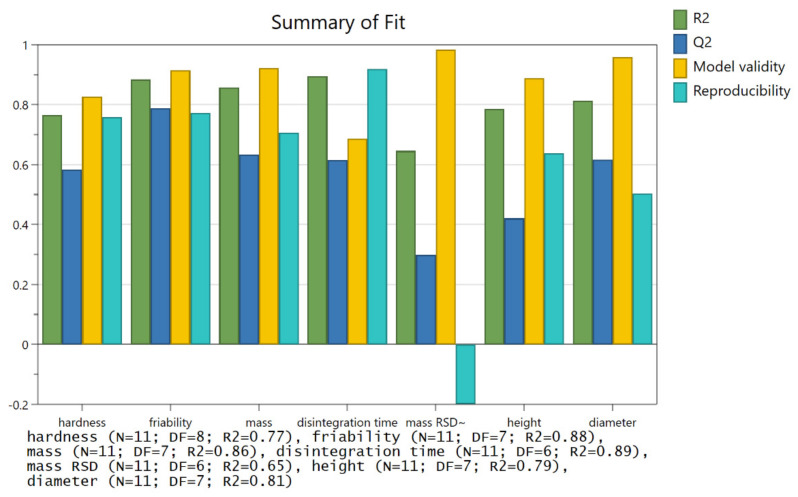
Summary of fit plot for the screening phase of DoE.

**Figure A2:**
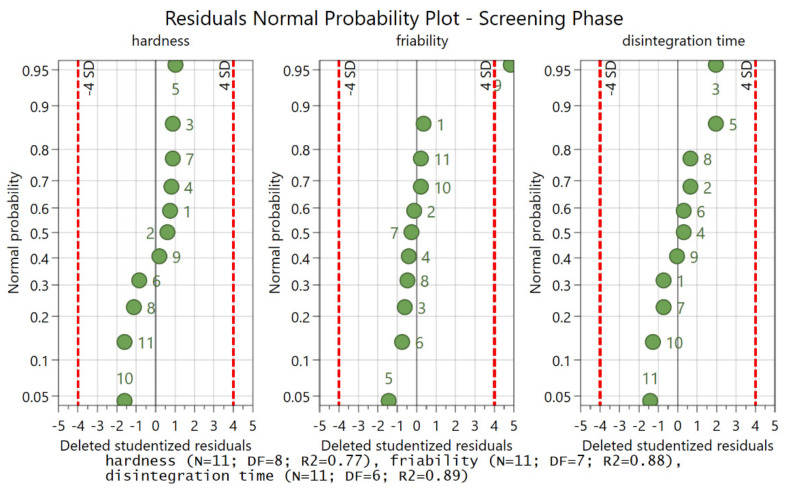
Normal probability plot showing the normal distribution of results for hardness, friability, and disintegration time of tablets during the screening phase of the DoE study.

**Figure A3:**
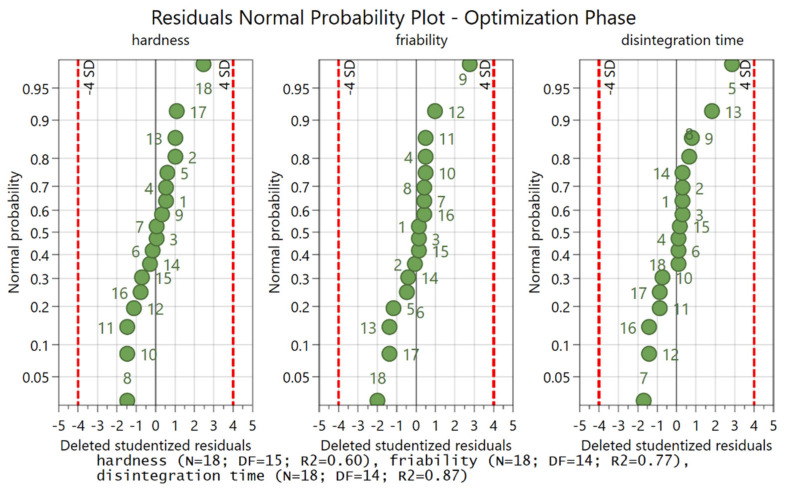
Normal probability plot showing the normality of distribution of results for responses hardness, friability, and disintegration time of tablets during the optimization phase of the DoE study.

As binder jetted tablets can be very fragile, it is important to optimize the process parameters and circumvent this issue to the greatest extent possible. To better understand the printing process and the tablet properties, five process parameters were analyzed in a screening phase of the DoE study: layer height, saturation parameter (number of active nozzles), doser speed, spreader speed, and printhead speed. Several responses were examined: disintegration time, hardness, and friability from the fragility point of view and mass, mass uniformity, tablet height, and tablet diameter as general quality properties of the tablets. The results for all experiments are summarized in Table 5. The results of all responses followed the normal distribution apart from hardness, disintegration time, and height, where a logarithmic transformation of results in the optimization phase was employed (Figure A2 and Figure A3). The model equations for the screening phase of the DoE study for hardness, friability, and disintegration time are shown in Equations (A1)–(A3). Well-fitting models with rather good predictability (Q^2^ values relatively close to R^2^) were obtained for almost all responses (Figure 2) with the exception of mass variation and tablet height models. Variations in the mechanical properties of the tablets were well explained with the models (hardness: R^2^ = 0.77, friability: R^2^ = 0.88, disintegration time R^2^ = 0.89). Good models were obtained for general tablet quality properties as well (mass: R^2^ = 0.86, height: R^2^ = 0.79, diameter: R^2^ = 0.81). A poor model was created only for mass uniformity with R^2^ of 0.65 and negative reproducibility. A formulation optimization is necessary to further inspect the mass uniformity parameter, as change in the powder blend composition could lead to a better result. It is also possible that none of the printing process parameters impacts tablet mass uniformity.

**Figure A4:**
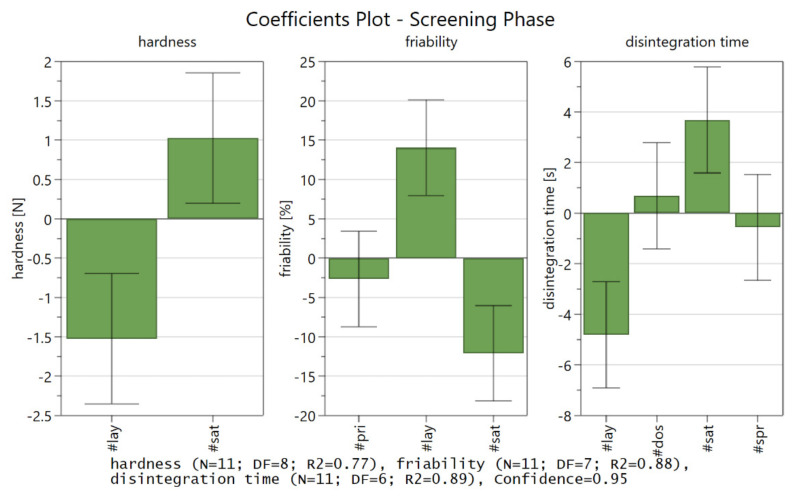
Coefficients plot for the screening phase of the DoE study which shows the influence of layer height and the saturation parameter on the mechanical properties of tablets, represented by responses hardness, friability, and disintegration time; where “lay” is layer height, “sat” is saturation, “pri” is printhead speed, “dos” is doser speed, and “spr” is spreader speed. In case the error bar crosses the line at 0, that parameter is not relevant and does not impact the response significantly.

Effects plots of individual response models from the screening study revealed that doser speed, spreader speed, and printhead speed did not have any major influence over the final tablet properties. This was a desirable outcome, proving the printing process very robust for these particular process parameters changes. Despite the speed of dosing and spreading, the mass of tablets was not influenced, as long as the powder bed was sufficiently filled during wiper passage. Any excessive powder was repeatably removed from the fabrication platform regardless of the selected spreader speed. In addition, fast movements of the printhead did not lead to any dimension inaccuracies or inclined droplet application that would lead to skewed tablet shape. An exception is the slight effect of printhead speed on mass uniformity; however, due to the poor model of this particular response, this influence is questionable and was not further investigated. Effects plots had on the other hand repeatedly demonstrated that layer height and saturation parameter have a profound effect on all measured tablet properties apart from tablet mass uniformity (Figure A4). This result was also confirmed in previous studies and was expected [14,21]. Yet the absence of impact of other printing parameters was not researched before.

The saturation parameter is defined as the percentage of active nozzles in the printhead. The liquid ink at least partially dissolves the solid binder within the powder blend. The higher the saturation parameter, the more binding liquid is applied through the printhead, resulting in higher powder bed saturation, stronger tablets, and easier handling due to the higher number of tight connections and solid bridges between particles after drying. Tablet hardness was the highest, disintegration time the longest, and friability the lowest when the binding liquid was jetted through all nozzles (100% saturation). However, each layer was extensively wetted as the binding fluid spread vertically and horizontally into the powder bed. As a result, the tablet height, diameter, and mass were larger then intended. The ink did not remain within layers but spread into the neighboring regions at the active printing area edges and the bottom of tablets. The reason for this wicking phenomenon might be the high ink wetting capacity of the water-soluble lactose-based powder blend and a local overapplication of the ink. The inclusion of an insoluble filler, such as anorganic phosphate salt, might improve this issue. Once the saturation parameter was reduced to 70%, the amount of applied ink was insufficient in the case of higher layer height (250 µm), which resulted in inadequate mechanical strength of tablets. In these instances, the solid binder did not dissolve to a sufficient extent. While tablets were successfully formed, tablets mechanical properties were poor for handling purposes. On the other hand, tablet dimensions slightly undershot the designed tablet model, as no excessive liquid permeated into the powder bed.

Layer height defines the thickness of each deposited powder layer. Particles can be spread into thin layers, as thin as the size of the D90 of the powder blend. After each powder layer is deposited, regardless of its thickness, the printhead applies the binding fluid. For this study, layer heights between 150 and 250 µm were selected. Lower layer heights provided tablets with better mechanical properties. Higher hardness, lower friability, and longer disintegration times were measured for these tablet samples. At lower layer heights, the printhead jets more ink per tablet, as more layers are needed to achieve the same tablet height. Therefore, more solid binder is dissolved, and particles of the dry tablet are better bound together. In addition, the binding liquid vertically penetrates a shorter distance to wet two subsequent layers together when lower layer heights are selected, and this leads to better interlayer linkage. The influence of layer height on tablet dimensions and mass was also noted. Similar to high saturation, larger and heavier tablets were produced at lower layer height. It could be that the layer was too thin to uptake all the applied ink. Excess fluid again spread into the unbound powder at the edges and the bottom of the tablets. At higher layer heights, this problem did not occur as dimensions were close to the designed model. However, the mechanical properties were poorer.

**Figure 3:**
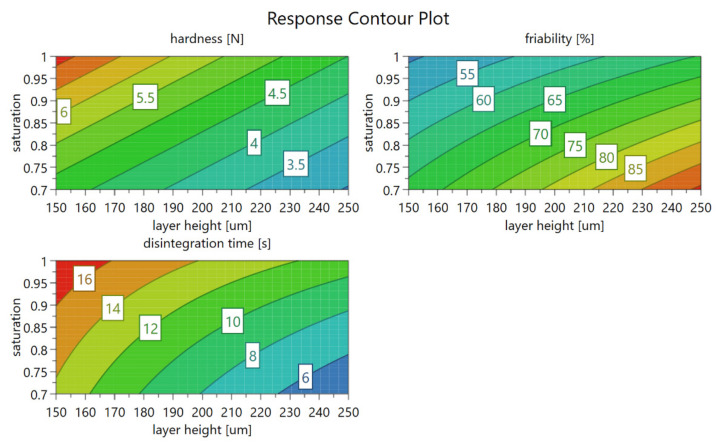
Response contour plot revealing the influence of layer height and the saturation parameter on the mechanical properties of tablets.

**Figure A5:**
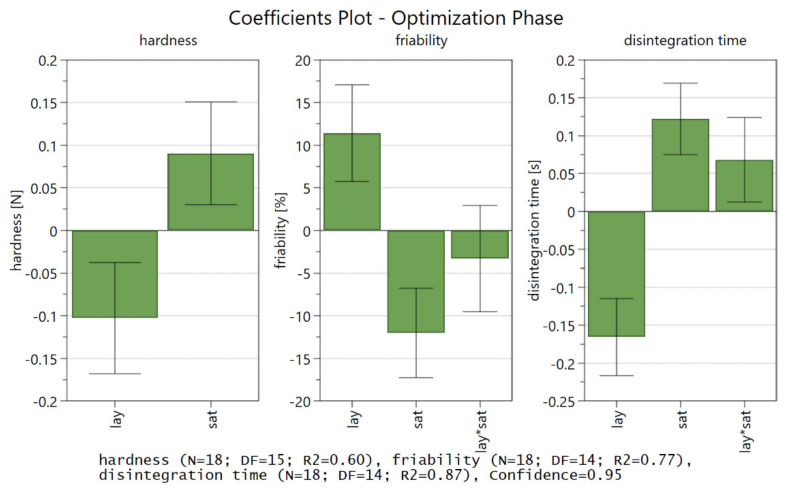
Coefficients plot for the optimization phase of the DoE study which shows the influence of layer height, saturation, and interactions on the mechanical properties of tablets, represented by responses hardness, friability, and disintegration time; where “lay” is layer height, “sat” is saturation and “lay*sat” is an interaction between the layer height and saturation. In case the error bar of a parameter crosses the line at 0, that parameter is not relevant and does not impact the response significantly.

Based on the obtained results, the layer height and saturation parameters were further examined in the experimental optimization phase for deeper insight into tablet properties and potential interactions. Model equations for the optimization phase of the DoE study for hardness, friability, and disintegration time are shown in Equations (A4)–(A6). A D-optimized approach was selected with seven additional experiments (Figure A5). Established models demonstrated similar R^2^ values for tablet disintegration times and friability but diminished in the case of tablet hardness (R^2^ = 0.60), although discrepancies between R^2^ and Q^2^ values decreased in all three cases. Based on established response surface models for tablet hardness, disintegration time and friability contour plots were created with a denoted range of achievable tablet mechanical properties (Figure 3). By varying the saturation parameter and layer height, we can achieve tablet strength between 3 and 8 N, friability between 50 and 90%, and disintegration time between 6 and 16 s for the selected formulation. This could be improved by choosing a better solid binder, such as PVP F 90.

**Figure 4:**
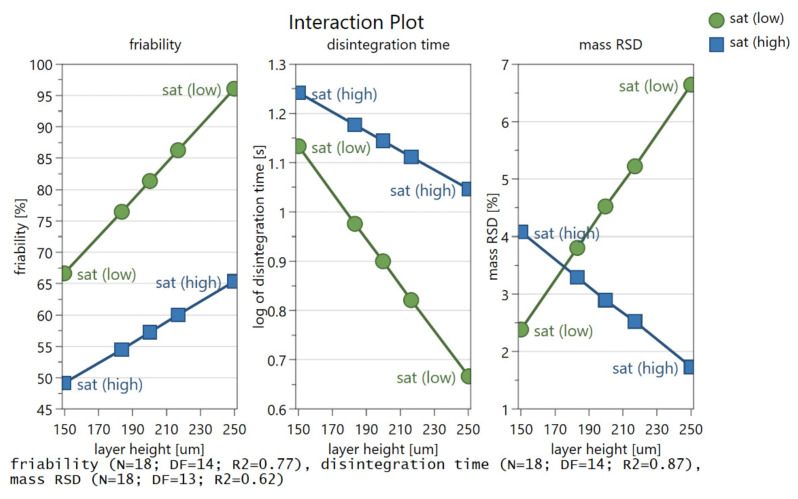
Interaction plot between layer height and saturation parameter for friability (**left**), disintegration time (**middle**) and mass uniformity (**right**).

Three interactions between saturation and layer height were determined, which support previous explanations with regard to tablet qualities (Figure 4). The first and second interaction are related in mechanism, and impact friability and disintegration time. It appears that when the saturation parameter is set to high values, layer height has less impact on friability and disintegration time, as the particles are well interlinked in the abundance of binder liquid. However, at low values of the saturation parameter, layer height becomes more important for mechanical properties. When saturation is set to low values, changes in layer height settings led to more pronounced variations in disintegration time and friability. This can be expected, as not enough solid binder is dissolved to bind the particles into a strong compact, i.e., especially at greater layer values. Therefore, a slight change in layer thickness can already result in considerable influence on friability and disintegration time.

The other interaction is related to mass uniformity. At low layer height settings, the difference in mass uniformity for both saturation levels is low in comparison with tablet mass uniformity difference for both saturation levels at high layer height settings. At higher layer heights, the number of active nozzles becomes important. While a high saturation parameter value even slightly improves mass uniformity, low saturation greatly worsens it. This could again be explained by poor particle binding due to a combination of high layer height and low saturation. Friable tablets can lead to occasional chipping of certain parts of the tablets during handling, contributing to poor mass uniformity. When layer height and saturation settings lead to tablets with poor mechanical properties (friability, disintegration time) tablet mass uniformity deviates from expected values.

### 3.4. Influence of Binding Fluid on Tablet Properties

**Figure 5:**
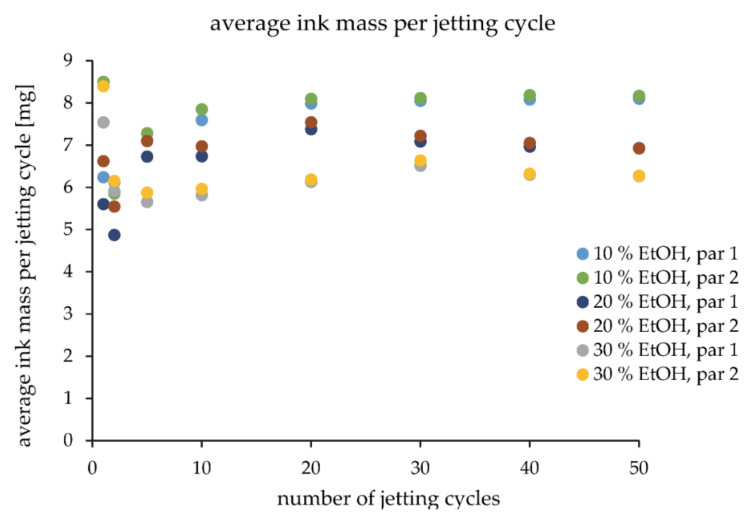
Average ink mass per jetting cycle over many layers. Par 1 and par 2 denote two parallel experiments to check for repeatability of measurements.

Apart from process parameters, binding fluid composition might also affect tablet properties. 10, 20, and 30% ethanol-water mixtures were used for this study. We hypothesized that the strongest compacts would form for the binding fluid with the highest product of the γL × cosΘ [24], the 10% ethanol-water mixture. Before this could be tested, the volume of the applied inks had to be standardized for all three binding fluids via the saturation parameter. Due to the different characteristics of the binding fluids, the printhead does not form droplets with identical volume or mass (Figure 5). The ink was collected after several jetting cycles to calculate an average mass of applied binding fluid per jetting cycle. This was done to assess the volume of each deposited droplet for all the inks and to better understand the jetting process over time. Measurements before 20 jetting cycles are less accurate due to potential ethanol evaporation and lower applied mass, leading to more experimental error and variability. However, we cannot omit the possibility that nozzle-driven formation of droplets is not quite repeatable from one instance to another. This can be argued by the fact that the Ohnesorge number value is too low, especially for 10 and 20% ethanol-water mixtures. After 40 jetting cycles, the difference in average ink mass per jetting cycle between two replicates became minimal. The fastest convergence of replicate results is observed for 30% ethanol-water mixtures. Droplet volume calculations were performed on data collected after 50 jetting cycles as this was deemed most precise. In addition, the average ink mass per jetting cycle remains uniform after 30 jetting cycles, proving stable average droplet deposition. Droplet volume for the 10% ethanol-water mixture was calculated at 4.3 nL, for the 20% ethanol-water mixture at 3.7 nL, and for the 30% ethanol-water mixture at 3.4 nL. The saturation level for the 20% ethanol-water mixture was corrected for a factor of 0.92 and for the 10% ethanol-water mixture for a factor of 0.80. Using these numbers as saturation parameter values, the volume of applied droplets was standardized for all inks.

**Figure 6:**
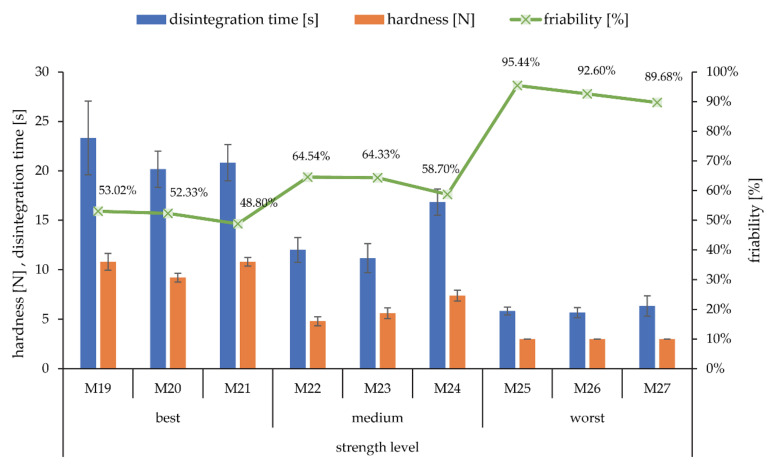
Influence of ink composition on disintegration time, hardness, and friability of tablets. Three strength levels of tablets (best, medium, worst) were determined by setting layer height and saturation accordingly. Within each strength level, tablets were prepared with the 10, 20, and 30% ethanol-water mixture.

The influence of ethanol content in ink was tested at three different tablet strength levels, which were modulated by the layer height and saturation parameter values: best, medium, and worst mechanical properties. It appears that the amount of ethanol content did not influence the tablet properties within the same tablet strength level (Figure 6). Process parameters settings in a DoE experimental set impacted the tablet qualities to a much larger extent. The 10% ethanol-water mixture did not produce compacts that were any stronger despite the theoretical increased strength of liquid bridges. In certain cases, the 30% ethanol-water mixture even generated tablets with slightly better mechanical properties. Friability was slightly lower for tablets prepared with the 30% ethanol-water mixture at all three levels compared to the 10% and 20% ethanol-water mixtures, which is contrary to the initial hypothesis. At the medium tablet strength level, hardness and disintegration time were also higher and friability lower for the 30% ethanol-water mixture. The same is true for tablet dimensions and mass. No definitive conclusions could be reached as it appears that ink composition in the studied range of properties does not specifically contribute to any properties of tablets if enough fluid is applied to dissolve the solid binder. However, this might not be the case for complex binding fluids which include a liquid binder or other insoluble excipients. Other papers have reached contrary conclusions on this topic [11,20], and further research is needed to clarify which liquid properties may affect tablet qualities. It could also be that there was an interplay present between the binder liquid properties that affect the maximum capillary pressure and at the same time affect the processability of the ink via the Ohnesorge number. The 30% ethanol-water mixture is just within the acceptable interval while the 10% and 20% ethanol-water mixtures fall below the 0.1 limit. Repeatable droplet deposition in a layer-by-layer fashion certainly affects uniformity of structure and therefore mechanical strength of these low-strength agglomerates with defined shape and size.

### 3.5. Assessing Powder Formulation Reuse Suitability

**Figure 7:**
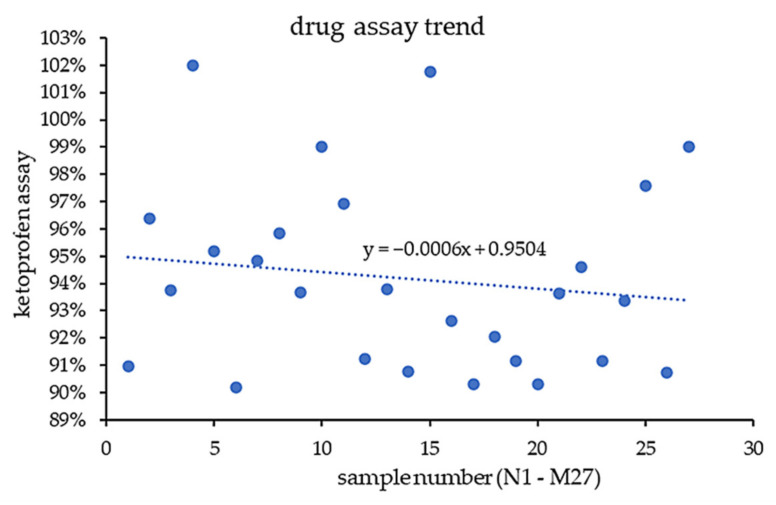
Loss of ketoprofen content in tablets throughout the experiments.

Throughout the experiments, the same powder blend with ketoprofen was used. After each tablet batch was prepared, the powder blend was collected and recycled for new batches to avoid unnecessary and unacceptable material waste. However, this approach has the potential for API loss or particle segregation. By measuring the ketoprofen content in tablets after each printing cycle, we wanted to confirm the suitability of powder blend reuse. To prevent API segregation, ketoprofen was already sieved in advance to match the particle size of other materials. Ketoprofen content was determined in the range between 90% and 102% (Figure 7). However, after 27 experiments, the trendline demonstrated that average ketoprofen content decreased only slightly from around 95% to 93%, proving that overall, ketoprofen content remains very similar. A slight loss of API is observed, but still within satisfactory limits. Based on this data, powder blend recycling for subsequent batches can be employed as long as the particle size of the powder blend components are similar. This might be a useful approach for a scaled binder jetting process, since the unbound powder mixture would have to be reused in a wasteless manner.

## 4. Conclusions

Binder jetting can be seen as a rather complicated manufacturing process for preparing 3D-printed tablets with poor mechanical properties and consequential difficulties in handling. Many authors have discussed the applicability of several powder blends to improve tablet performance by reducing the fragility of prepared tablets. However, the printing parameters are largely overlooked, despite providing an interesting alternative to modifying tablet properties. A model printable formulation with ketoprofen was proposed to better understand and improve the printing process. Apart from the particle size distribution of the powder blend, it was found that flow properties should also be just right (with an HR between 1.26 and 1.37). On a custom-made 3D printer Picojet D220, five printing parameters were tested and their influence on the properties of tablets elucidated by employing the DoE approach. Only the saturation parameter (number of active nozzles) and layer height were found to significantly influence tablet properties. Tablet disintegration time, friability, and hardness can be changed two-fold by adjusting the two printing parameters. Increasing the saturation parameter and reducing layer height provided tablets with the best mechanical properties. However, this combination yielded edge over-wetting and consequently tablets with larger dimensions and mass due to unwanted spreading of the binder liquid into the neighboring regions of the powder bed. This issue can most probably be mitigated with the addition of insoluble fillers to the powder blend or by adjusting the layer height and local saturation parameter value. In this way, the applied binding liquid would remain within the envisaged boundaries of the tablet model. However, further experimental work in this direction is needed.

The impact of the ink’s composition on the mechanical properties of tablets was also evaluated. In the tablet printing process, 10, 20, and 30% ethanol-water mixtures were used. Based on the highest product of the γL × cosΘ, denoting the strongest liquid bridges between particles, our initial hypothesis presumed the best mechanical properties for tablets with the 10% ethanol-water mixture. This was not the case, as a correlation between the amount of ethanol in the binder liquid and tablet properties was not found. Process parameters influence tablet properties to a much greater extent.

Lastly, API content was measured for every batch of the produced tablets. Since the initial powder blend was reused for all 27 experiments, potential ketoprofen segregation and/or loss might occur. The suitability of powder blend recycling was confirmed, as overall only a slight decrease of ketoprofen content was observed. As the particle size of all powder blend components was similar, segregation was not likely to occur. In the pursuit of a 3D binder jetted tablet with further improved mechanical properties, current findings will be complemented in future research with formulation optimization.

## Figures and Tables

**Table 1:** Process parameters settings for each DoE run.

Run	Printhead Speed [mm/s]	Layer Height [µm]	Doser Speed [mm/s]	Saturation Level	Spreader Speed [mm/s]
N1	60	150	3	1	40
N2	120	150	3	0.7	20
N3	60	250	3	0.7	40
N4	120	250	3	1	20
N5	60	150	4	1	20
N6	120	150	4	0.7	40
N7	60	250	4	0.7	20
N8	120	250	4	1	40
N9	90	200	3.5	0.85	30
N10	90	200	3.5	0.85	30
N11	90	200	3.5	0.85	30
C12	90	150	3.5	0.9	30
C13	90	250	3.5	0.8	30
C14	90	183	3.5	0.7	30
C15	90	217	3.5	0.7	30
C16	90	183	3.5	1	30
C17	90	217	3.5	1	30
C18	90	200	3.5	0.85	30

**Table 2:** Process parameters settings for ink composition runs.

Run	Layer Height [µm]	Saturation Level	Binding Fluid [m/m % EtOH]	Tablet Strength Level
M19	150	0.80	10	high strength
M20	150	0.92	20	high strength
M21	150	1.00	30	high strength
M22	200	0.68	10	medium strength
M23	200	0.78	20	medium strength
M24	200	0.85	30	medium strength
M25	250	0.56	10	low strength
M26	250	0.64	20	low strength
M27	250	0.70	30	low strength

**Table 3:** Powder blend composition and characterization.

Powder Blend	Substance	Composition [%]	Flowability		Particle Size		
			Carr Index	Hausner Ratio	D10 [µm]	D50 [µm]	D90 [µm]
Z1	Pharmatose^®^ 200M	69.5	40	1.68	6.1	43.8	111.0
	Ketoprofen	20.0			7.0	92.9	182.0
	Plasdone^®^ K-25	10.0			30.3	74.3	140.0
	Syloid^®^ 244 FP	0.5			1.7	3.9	14.4
Z2	Avicel PH-101^®^	69.5	33	1.50	22.0	58.0	124.0
	Ketoprofen	20.0			7.0	92.9	182.0
	Plasdone^®^ K-25	10.0			30.3	74.3	140.0
	Syloid^®^ 244 FP	0.5			1.7	3.9	14.4
Z3	Pharmatose^®^ 125M	69.5	27	1.37	38.4	85.2	152.0
	Ketoprofen	20.0			7.0	92.9	182.0
	Plasdone^®^ K-25	10.0			30.3	74.3	140.0
	Syloid^®^ 244 FP	0.5			1.7	3.9	14.4
Z4	Tabletosse^®^ 70	69.5	20	1.25	103.0	192.0	327.0
	Ketoprofen	20.0			7.0	92.9	182.0
	Plasdone^®^ K-25	10.0			30.3	74.3	140.0
	Syloid^®^ 244 FP	0.5			1.7	3.9	14.4
Z5	Supertab^®^ 14 SD	69.5	19	1.23	59.4	127.0	228.0
	Ketoprofen	20.0			7.0	92.9	182.0
	Plasdone^®^ K-25	10.0			30.3	74.3	140.0
	Syloid^®^ 244 FP	0.5			1.7	3.9	14.4
Z6	Pearlitol^®^ 100 SD	69.5	17	1.21	66.3	105.0	165.0
	Ketoprofen	20.0			7.0	92.9	182.0
	Plasdone^®^ K-25	10.0			30.3	74.3	140.0
	Syloid^®^ 244 FP	0.5			1.7	3.9	14.4
Z7	Pharmatose^®^ 125M	34.75	21	1.26	38.4	85.2	152.0
	Pearlitol^®^ 100 SD	34.75			66.3	105.0	165.0
	Ketoprofen	20.0			7.0	92.9	182.0
	Plasdone^®^ K-25	10.0			30.3	74.3	140.0
	Syloid^®^ 244 FP	0.5			1.7	3.9	14.4

**Table 4:** Binding fluid composition and characterization.

Binding Fluid [m/m % EtOH]	Density [g/mL]	Viscosity [mPas]	Surface Tension [mN/m]	Ohnesorge Number	Contact Angle [°]	γL × cosΘ
10%	0.9815	1.4	46.80	0.046	31.7	39.82
20%	0.9672	1.6	38.22	0.059	27.0	34.05
30%	0.9500	2.2	32.74	0.088	19.3	30.90
40%	0.9300	2.4	29.69	0.102	16.0	28.54
10% + 10% PEG	0.9984	2.9	46.31	0.095	30.7	39.82
20% + 10% PEG	0.9887	3.6	38.38	0.131	27.8	33.95
30% + 10% PEG	0.9709	4.2	33.12	0.166	19.3	31.26
40% + 10% PEG	0.9495	4.2	30.04	0.176	16.1	28.86

**Table 5:** Summary of results for all responses in the DoE study.

Run	Mass [mg]	Diameter [mm]	Height [mm]	Hardness [N]	Disintegration Time [s]	Friability [%]
N1	633.9 ± 20.9	15.94 ± 0.52	7.01 ± 0.42	8 ± 1	18 ± 2	50.42
N2	479.0 ± 10.4	15.36 ± 0.25	5.53 ± 0.11	6 ± 0	14 ± 1	66.26
N3	370.1 ± 29.7	14.40 ± 0.19	4.31 ± 0.18	3 ± 0	5 ± 1	97.24
N4	468.7 ± 12.8	15.05 ± 0.16	5.29 ± 0.07	5 ± 0	11 ± 2	68.72
N5	503,6 ± 15,7	15,25 ± 0,16	5.50 ± 0.19	8 ± 1	24 ± 1	41.15
N6	473.7 ± 8.5	15.14 ± 0.18	5.36 ± 0.96	5 ± 1	14 ± 0	63.11
N7	347.6 ± 18.3	14.30 ± 0.18	4.31 ± 0.13	3 ± 0	4 ± 1	98.90
N8	440.1 ± 5.4	14.85 ± 0.29	5.12 ± 0.09	3 ± 1	12 ± 0	68.35
N9	509.8 ± 23.6	15.23 ± 0.21	5.70 ± 0.23	5 ± 0	12 ± 4	88.05
N10	438.8 ± 8.9	14.81 ± 0.22	4.98 ± 0.07	3 ± 1	9 ± 1	73.26
N11	439.2 ± 5.2	14.57 ± 0.18	4.89 ± 0.15	3 ± 1	9 ± 1	73.43
C12	503.9 ± 16.9	15.37 ± 0.31	5.32 ± 0.24	5 ± 0	13 ± 1	62.68
C13	419.1 ± 12.3	14.85 ± 0.21	5.03 ± 0.19	4 ± 0	8 ± 2	75.49
C14	297.6 ± 12.4	14.42 ± 0.18	4.76 ± 0.11	4 ± 0	10 ± 1	73.10
C15	352.6 ± 21.2	14.34 ± 0.14	4.63 ± 0.32	3 ± 0	7 ± 2	87.38
C16	430.5 ± 24.3	15.04 ± 0.09	5.15 ± 0.10	5 ± 0	12 ± 1	57.80
C17	447.7 ± 4.9	14.97 ± 0.16	5.12 ± 0.06	7 ± 1	11 ± 1	49.35
C18	448.0 ± 7.4	15.02 ± 0.13	5.23 ± 0.07	7 ± 1	11 ± 1	54.22

## Data Availability

The data presented in this study are contained within the article.

## Appendix B

Equation (A1). Model equation for parameter hardness after the screening phase of the DoE study, where H is hardness, lay is layer height, and sat is saturation.

(A1) H=4.800 − 1.525 × lay+1.025 × sat

Equation (A2). Model equation for parameter friability after the screening phase of the DoE study, where F is friability, pri is printing speed, lay is layer height, and sat is saturation.

(A2) F=71.717 − 2.659 × pri+14.034 × lay − 12.109 × sat

Equation (A3). Model equation for parameter disintegration time after the screening phase of the DoE study, where D is disintegration time, lay is layer height, dos is doser speed, sat is saturation, and spr is spreader speed.

(A3) D=12.073 − 4.813 × lay+0.688 × dos+3.688 × sat − 0.563 × spr

Equation (A4). Model equation for parameter hardness after the optimization phase of the DoE study, where H is hardness, lay is layer height, and sat is saturation.

(A4) H=0.665 − 0.103 × lay+0.090 × sat

Equation (A5). Model equation for parameter friability after the optimization phase of the DoE study, where F is friability, lay is layer height, sat is saturation, and lay*sat is an interaction parameter between layer height and saturation.

(A5) F=69.261+11.395 × lay − 12.017 × sat − 3.315 × lay*sat

Equation (A6). Model equation for parameter disintegration time after the optimization phase of the DoE study, where D is disintegration time, lay is layer height, sat is saturation, and lay*sat is an interaction parameter between layer height and saturation.

(A6) D=1.021 − 0.166 × lay+0.122 × sat+0.068 × lay*sat
